# A Mechanism-based Perspective on the Use of Flavonoids in the Treatment of Diabetes and its Complications

**DOI:** 10.2174/0115733998335480241022084655

**Published:** 2024-11-29

**Authors:** Sandesh Kumar Pattanaik, Manisha Prajapati Anil, Sudipta Jena, Diptirani Rath

**Affiliations:** 1 School of Pharmaceutical Sciences, Siksha ‘O’ Anusandhan Deemed to be University, Bhubaneswar, Odisha, 751003, India;; 2 School of Pharmacy, Sharda University, Knowledge Park-3, Greater Noida, U.P., 201306, India;; 3 Centre of Biotechnology, Siksha ‘O’ Anusandhan Deemed to be University, Bhubaneswar, Odisha, 751003, India

**Keywords:** Flavones, flavanones, flavonols, flavanonols, type 1 diabetes, type 2 diabetes, gestational diabetes

## Abstract

Diabetes is a chronic, irreversible, non-infectious metabolic syndrome associated with low insulin production by the pancreas or due to insulin resistance. The management landscape for diabetes is swiftly evolving due to ongoing advancements. Conventional treatment approaches have struggled to fully address the root causes of the disease while also carrying significant risks of adverse effects. Flavonoids are an extensive class of phytonutrients present in grains, vegetables, fruits, cocoa, tea, wine, and nuts. Many studies have reported that flavonoids have shown diversified pharmacological activity in recent years. Thus, this review will give you an overview of the significant anti-diabetic potential of promising flavonoids. Various search engines such as PubMed, Scopus, Google Scholar, and WoS have been explored by using the keywords “apigenin,” “luteolin,” “naringenin,” “hesperidin,” “kaempferol,” “quercetin,” “myricetin” and “taxifolin” with “anti-diabetic.” The anti-diabetic activity of flavonoids is attributed to various mechanisms, including α glucosidase, α-amylase inhibitory effects, GLUT4 expression, antioxidant, and apoptosis. However, their inadequate biopharmaceutical qualities make their effectiveness in clinical translation constrained. This review aims to highlight plant-derived flavonoids through *in-vitro*, *in vivo*, and clinical insights. Additionally, the review highlights the recent advancement in the drug delivery system in diabetes to overcome the limitation of flavonoids.

## 1 INTRODUCTION

Diabetes is a long-term metabolic condition characterized by insufficient insulin synthesis by the pancreas or inadequate utilization of insulin by the body, according to the World Health Organization (WHO). Diabetes has emerged as the third leading cause of death worldwide, following cancer and cardiovascular disease, owing to its rising incidence and mortality rates among the global population [1].

According to the most recent data from the International Diabetes Atlas 10^th^ edition, the prevalence of diabetes is expected to be 537 million people worldwide in 2021, with the majority of those affected in low and middle-income countries. By 2045, that number is expected to climb to 46%. The epidemiology study proposed that the regions with the highest prevalence and increasing risk factors for diabetes were Africa, Western/Eastern regions, and Europe [2]. In regions like India, the burden of diabetes has increased in the past decades, and it is estimated to increase by 69.9 million people with diabetes prevalence by 2025 [3].

Metabolic disorder term refers to any physiological process that interferes with normal metabolic function. These often have slow progression and prolonged duration compared to other metabolic syndromes. The natural metabolism of glucose, the body's main energy source, becomes impaired by individuals with diabetes. There are three types of diabetes: Type-I diabetes (TIDM), Type-II (TIIDM), and gestational diabetes (GD). TIDM is a cell-mediated hypersensitivity reaction, where T cell attacks the own pancreatic β-cells, impeding insulin production [4]. TIIDM diabetes is the most prevalent form of the disease globally, accounting for approximately 90% of all diagnosed cases [5]. In TIIDM, diabetes is acquired by insulin resistance; the “body doesn’t respond to insulin.” GD, also known as maternal diabetes, is identified during pregnancy. It is typified by elevated blood sugar levels that arise in a pregnant woman who has never had diabetes before. This condition, known as gestational diabetes, normally occurs around the 24^th^ week of pregnancy and usually resolves after childbirth. In this condition, the body fails to generate sufficient insulin to fulfill the additional demands of pregnancy. Consequently, blood sugar levels may elevate beyond the usual range [6, 7].

Throughout the decades, people have used plants as the primary source of mitigating various disease elements. Plants have primary and secondary metabolites [8]. The metabolic pathway produces the plant’s secondary metabolites and has a wide range of impacts on the plant itself and other living organisms [9,10]. Much research has revealed that the plant secondary metabolites exhibit various pharmacological activities such as anti-diabetic [11], antiarrhythmic [12], anti-hypertensive [13], anti-ulcer [14], anti-malarial [15], anti-cancer [16], analgesic [17], anti-spasmodic [18], anti-microbial effects [19], multiple sclerosis [20] and neuroprotective effect [21]. Till now, 200000 diverse secondary metabolites have been identified, and research is ongoing to investigate new metabolites with unique pharmacological roles [22].

The plant's secondary metabolites are broadly classified according to their chemical structure, such as alkaloids, saponin, glycoside, flavonoids, and terpenoids [23]. Flavonoids, which belong to the polyphenolic chemicals class, have been demonstrated in clinical studies to have therapeutic effects in the treatment of a variety of disorders. Over 10,000 flavonoids have been found from various biological sources [24]. Findings have shown that flavonoids effectively inhibit diabetes [25], obesity [26], pulmonary arterial hypertension [27], cancer [28], myocardial infarction [29], atherosclerosis [30], chronic obstructive pulmonary disease [31], glaucoma [32], gouty arthritis [33], and anti-inflammatory diseases [34].

Flavonoids have different subgroups based on their chemical structure: anthocyanidins, anthoxanthin, chalcone, flavanone, flavanonols, flavans, isoflavonoids, and neoflavonoids [35]. In both animal and cellular model systems, it has been demonstrated that many of these flavonoids display promising anti-diabetic potential [36]. The anti-diabetic potential of flavonoids may be due to their modulation in the key glucose metabolic pathways and antioxidant properties. The presence of functional groups like methoxy (OCH_3_) and glycosyl groups improved the biological activity and efficiency of flavonoids in modulating glucose metabolism, and the presence of hydroxyl group (-OH) enabled the antioxidant capabilities, which interfered with the various glucose metabolism receptors such as PPAR-γ and AMPK [37, 38].

This present review aims to highlight the plant-derived flavonoids, specifically apigenin, luteolin, naringenin, hesperidin, kaempferol, quercetin, myricetin, and taxifolin in the treatment of diabetes through *in-vitro*, *in-vivo*, and clinical insights. Additionally, the review highlights the recent advancement in the drug delivery system in diabetes to overcome the limitation of flavonoids (Fig. **1**).

## 2 PATHOGENESIS OF DIABETES

### 2.1 Insulin

Insulin is a polypeptide hormone secreted by the pancreatic β-cells of islets of Langerhans. It is a hormone consisting of fifty-one amino acids that serve a crucial part in preserving glucose homeostasis through facilitating cellular utilization of glucose, moderating carbohydrate, protein, and lipid metabolism, and proliferation *via* mitogenic actions and encouraging cell division [39]. Insulin is often produced in response to glucose ingestion, which involves metabolic breakdowns of consumed glucose and intracellular absorption, known as glucose-induced insulin stimulation. It is a primary carrier for transporting internal glucose to the insulin-dependent tissue and cells, liver, and muscles for energy production and storage [40]. The liver is one of insulin's main targets; it stimulates the synthesis of glycogen and fatty acids from glucose. Studies suggested that glycolysis, pentose-phosphate shunt, glucuronic acid pathway glycogenesis, and gluconeogenesis utilize the upregulation of glucose [41]. The enzyme glucokinase is mainly responsible for the phosphorylation of glucose that relates to insulin secretion. The ailment in the enzyme glucokinase leads to insulin resistance and impaired glucose-mediated insulin release [42].

Insulin facilitates several metabolic pathways, such as PI3K/ Akt and Mitogen-activated protein kinase (MAPK), starting from binding to the Insulin receptor (IR) to mediate insulin receptor substrate (IRS). The activation of PI3k results in respectable inhibition of glycogen synthase and ATP citrate lyase activity. IRS-1, when bound to growth factor receptor bound (Grb2), results in the activation of Ras recruits c-Raf, which phosphorylates MAPK/Erk kinase. The MAPK/Erk phosphorylates Erk. Erk is translocated to the nucleus, which further promotes protein synthesis, cell division, and cell growth (Fig. **2**) [43].

### 2.2 Inflammatory Mediators

Inflammatory mediators, such as IL-6, IL-1, TNF-α, c-reactive protein, and chemokines, are involved in diabetes. These mediators generated one more pathophysiology of diabetes. In the case of obesity, different inflammatory mediators are released from the liver, β-cells of the pancreas, and tissues. TNF-α is generally released from visceral adipose tissue [44]. Inflammatory cytokines are released by adipose tissue in response to many conditions, including hypoxia. Several causes of released inflammatory mediators are discussed in the literature, but proper pathophysiology is unclear. Cholesterol causes induced inflammation *via* upregulating toll-like receptor pathways and nuclear factor-κB releases, TNF-α, other chemokines, and cytokines. The alteration of renin-angiotensin and gut microbiota is also responsible for inflammatory-mediated insulin resistance. Macrophages are also accountable in diabetes caused by inflammatory mediators as they emit cytokines [45, 46]. In adipocytes, these cytokines activate c-Jun N-terminal kinase (JNK), serine kinases IκB kinase β (IKKβ), mammalian target of rapamycin 32 (mTOR32), and ribosomal protein S6 kinase (S6K) that mediate the inhibitory phosphorylation of IRS1, resulting in insulin resistance [47].

### 2.3 Metabolites

#### 2.3.1 Amino Acids

Diabetes and amino acids are connected because amino acids are required to synthesize glucose from non-carbonate sources [48]. Premature diabetes progresses because of the overexpression of amino acids, which increases glucose synthesis. The dysfunctional insulin pathway has increased concentrations of branch-chain amino acids like valine and leucine. Higher levels of branched-chain amino acids are directly associated with diabetes and negatively associated with insulin sensitivity, according to cohort research data [49]. Leucine inhibited insulin receptor activity *via* activating the mTOR Complex 1 (mTORC1) pathway. Tyrosine and phenylalanine, two aromatic amino acids, are also linked to gluconeogenesis. After giving phenylalanine to the mice, scientists concluded that it causes insulin resistance [50]. Insulin resistance and several other changes are brought by tryptophan-kynurenine signaling in diabetic individuals. This route also alternated metabolic markers in prediabetic conditions [51]. The glycine receptor is found on the β islet pancreatic cells and is responsible for insulin sensitivity. Glycine deficiency results in inadequate synthesis of GSH, which cannot prevent the ensuing diabetic tissue damage [52]. Researchers administered a sucrose-rich diet to the rat. They concluded that it reduces phosphorylation of insulin receptors in serine residue and upregulates phosphorylation in tyrosine residue of beta subunit, leading to increased insulin sensitivity [53]. Asparagine and aspartate's role in diabetes is still unclear. Some studies reported that it is protective against diabetes, while some studies showed it is diabetogenic. The byproduct of asparagine, oxaloacetate, is used in glucose synthesis by the Kerbs cycle, so the overactivation of asparagine leads to the risk of diabetes. Asparagine and aspartate interchange and form glutamate, leading to diabetes risk. Asparagine upregulates the mTORC1 and decreases the phosphorylation of the AMPK pathway, which causes insulin resistance [54]. Some other studies are also present in the literature, which showed the positive effect of asparagine. Thus, further investigation is required for asparagine. Serine is another amino acid involved in the pathogenesis of diabetes by upregulating glucose tolerance, insulin secretion, and sensitivity [55].

#### 2.3.2 Fatty Acids

Fatty acids are lipid biomolecules comprising a chain of joined carbon atoms, usually with a carboxyl group (-COOH) at one end. They are fundamental building blocks for numerous biological processes in the body and are a central component of dietary fats and oils. Numerous components are involved in the biochemical mechanism that underlies the intricate interaction between fatty acids and the etiology of diabetes, specifically in regulating inflammation and energy homeostasis [56]. Fatty acids are involved in the pathophysiology of TIIDM and insulin resistance by influencing insulin receptor signaling, glucose transporter translocation, and cell membrane characteristics [57]. Various enzymes, such as 12 lipoxygenases, have a role in the metabolism of fatty acids, especially polyunsaturated fatty acids. These enzymes trigger both pro-inflammatory and anti-inflammatory lipid mediators. Dobrain has shown that 12-lipoxygenase regulates inflammation at various stages of diabetes progression, suggesting it could be a possible target for treatments [58]. Furthermore, neurons in the brain, namely in the hypothalamus, that are sensitive to fatty acids have a crucial function in regulating energy and glucose balance. This includes controlling food behaviour, insulin release, and its effects. The molecular agents responsible for the actions of fatty acids include ion channels and fatty acid translocator receptors CD36. Dysregulated metabolism-secretion coupling in pancreatic β-cells, characterized by reduced mitochondrial fatty acid oxidation and oxidative phosphorylation, contributes to decreased insulin secretion in diabetes. This dysregulation is associated with elevated reactive oxygen species (ROS) generation and attenuated cellular antioxidant defence systems [59, 60]. Endocannabinoids are synthesized from the fatty acids precursors, *i.e.*, AEA (anandamide) and 2AG (2 arachidonoylglycerol); this endocannabinoid system influences TIIDM by modulating the hypothalamus pituitary adrenal (HPA) axis. Abnormalities of the HPA axis, a critical regulator of energy metabolism, are associated with alterations in cannabinoid receptor expression and endocannabinoid hydrolase in the development of TIIDM. Moreover, a rise in non-esterified fatty acid (NEFA) levels is associated with β-cell dysfunction, insulin resistance, and apoptosis, and all factors contribute to TIIDM [61].

### 2.4 Mitochondrial Dysfunctions

#### 2.4.1 Oxidative Stress

Oxidative stress arises from a breakdown between the body's antioxidant defence system and the generation of ROS, which have the potential to harm proteins, cells, and DNA. The oxidative stress level is influenced by the free radicals that may be nitrogen or oxygen molecules, *i.e.*, hydroperoxyl, hydroxyl radicals, hydrogen peroxide, superoxide anion radical, alkoxyl radical, and peroxyl radical [62]. Studies indicate that oxidative stress plays a significant role in the etiology of diabetes [63]. Furthermore, the ROS systems activate stress-responsive pathways. The activation of glycolytic, hexosamine, protein kinase C, polyol, and advanced glycation end-product pathways also contribute to diabetes [64]. In addition, the ROS system disrupts insulin signaling pathways by activating different types of protein kinase C, boosting serine phosphorylation of insulin receptor substrate proteins, and blocking insulin receptor autophosphorylation. As a result of these processes, insulin cannot effectively target tissues like adipose, muscle, and liver. Pancreatic β cells with comparatively low antioxidant defense activity levels are especially vulnerable to oxidative stress. That leads to impaired insulin secretion, damages the β cells, and promotes apoptosis [65].

### 2.5 The Glucose Transporters

#### 2.5.1 GLUT-4

The GLUT-4 transporter is a primary transporter responsible for enhancing glucose absorption. It acts when insulin is present. Insulin increases the translocation of GLUT-4 in the skeletal muscle and adipose tissue. Elevated levels of glucose uptake can be attained by enhancing the sensitivity of GLUT-4. GLUT-4 enhances glucose absorption by a process triggered by insulin. Insulin binding to its receptor begins a signaling cascade that activates PI3K-Akt. Akt activation stimulates the fusion of GLUT-4 vesicles with the cell membrane, enabling glucose uptake by GLUT-4. Consequently, it enhances the presence of GLUT-4 in the membrane, leading to an increase in the absorption of blood glucose and the prevention of diabetes diseases. Insulin increases the 5 to 30-fold GLUT-4 in the membrane. These increases depend on three factors: increasing exocytosis (taking GLUT-4 from cytosol to plasma membrane), decreasing endocytosis (taking plasma membrane to cytosol), and rising GLUT-4 pool in the recycling membrane [66].

## 3 PROMISING DIABETIC TREATMENT WITH FLAVONOIDS

The anti-diabetic potential of flavonoids has been discussed, and the critical chemical structure of flavonoid compounds is shown in Fig. (**3**).

### 3.1 Apigenin

The emerging effect of apigenin as an anti-diabetic candidate is well documented in various *in-vitro* and *in-vivo* studies. The overall research is summarized in Table **1** of the anti-diabetic potential study of apigenin, which was conducted in an insulin-resistance HepG2 cell line. Through various assays and evaluations, such as measurements of cell viability, glucose intake, glycogen synthesis, and signaling pathway regulation, apigenin exhibits potential for reducing symptoms associated with diabetes. It increases the expression of necessary insulin signaling pathway components, GLUT4, IRS-1, and IRS-2, which improves glucose absorption and glycogen production. Also, apigenin lowers intracellular AGE and ROS levels, reducing diabetes-related oxidative stress and inflammation [67].

The protective action of apigenin reduces the endoplasmic reticulum (ER) stress-mediated cell death in INS-1 β- cells. According to the study, apigenin increases insulin secretion in INS-1D cells in concentration-dependent ways. This impact peaks at 30 µM and decreases at higher doses. Furthermore, as demonstrated by lower DNA fragmentation, fewer apoptotic cells seen under a microscope, and reduced production of apoptotic markers, including cleaved caspase-3 and CHOP, apigenin lessens ER stress-induced apoptosis. Apigenin suppresses the overexpression of thioredoxin-interacting protein (TXNIP), a mediator of β-cell death brought on by ER stress [68]. Furthermore, enzymatic assays were conducted in α-glucosidase from the plant extract of *Hyphaene thebaica*. The LC-HR-MS study shows more significant inhibitory activity than a thorough metabolic profile, indicating that apigenin might be involved in the plant's antidiabetic effects [69]. An Apigenin analog L22 showed a promising impact on TIIDM. Honghui Liu conducted a study both in *in-vitro* and *in-vivo*. The *in-vitro* model L22 compound is a potent α-glucosidase inhibitor, and the *in- vivo* model shows an exact protective effect in the liver and pancreas by reducing oxidative stress and confirming that it lowers the fasting blood glucose level and insulin resistance. The high-fat diet-STZ-induced diabetic mice were taken as the *in-vivo* model for this [70].

Apigenin was found to be a therapeutic benefit over its anti-diabetic effects by protecting against vascular dysfunction linked to diabetes and obesity. In a study, the author discovered that apigenin therapy enhanced endothelial function in high-glucose environments, decreased oxidative stress, and activated signaling pathways related to vascular health, such as the Nrf2/HO-1 and AMPK/PI3K/Akt/eNOS pathways. These findings were made using animal models, *ex-vivo,* and *in-vitro* tests. Furthermore, apigenin administration in obese mice increased glucose tolerance, lowered blood pressure, normalized lipid profiles, and enhanced vascular function, according to *in-vivo* studies [71]

The apigenin modulates CD3 and GLUT4 protein expressions. CM Hossain and team, in a study of induced diabetes compared with glipizide, showed that apigenin treatment dramatically decreased fasting blood glucose levels. According to histopathological research, apigenin lessened the harm associated with hyperglycemia by maintaining the cellular architecture of critical organs like the liver, kidneys, and pancreas in a normal range. In addition, apigenin administration decreased lipid peroxidation, increased the activity of antioxidant defense enzymes, and altered the activity of phase I and II drug-metabolizing enzymes. Notably, apigenin downregulated CD38 expression and boosted GLUT4 translocation in skeletal muscles, suggesting enhanced glucose homeostasis and possible defense against diabetes-related metabolic disorders [72].

Furthermore, the protective effect of apigenin-loaded solid lipid nanoparticles was investigated against the STZ-induced diabetic model; early *in-vitro* investigations showed that the SLNPs released Apigenin continuously for 24 hours. In the animal model, rats with diabetes that were given Apigenin-SLNPs had much lower blood glucose levels and higher insulin levels than the control groups. The anti-inflammatory and antioxidant properties of the Apigenin-SLNPs were shown through the restoration of superoxide dismutase, catalase, and malondialdehyde levels, as well as the downregulation of pro-inflammatory cytokine expression, such as interleukin-6, interleukin-1β, and tumor necrosis factor-alpha. Furthermore, histopathological analysis revealed that Apigenin-SLNPs reduced diabetic kidney damage. As a result, it indicates that apigenin modulates the Nrf2, NF-κB, and heme oxygenase signaling pathways and reduces the oxidative stress and inflammation associated with diabetes [73].

Cardiovascular complications are the most common and highly prominent in diabetes. In a study, the author set out to determine how diabetic mellitus affected the functional, histological, biochemical, and biochemical components of cardiovascular health in rats. The apigenin administration improves vital factors such as reduction in Drp1 expression, lipid profile modulation, blood pressure regulation, preservation of cardiac indicators, and repair of heart tissue architecture. These results imply that apigenin has therapeutic value in reducing the cardiovascular problems linked to TIIDM [74].

### 3.2 Luteolin

The anti-diabetic potential of luteolin was investigated through *in-vivo* and *in-vitro* models. The author discovered that luteolin prevented lipotoxicity-induced β cell death and preserved glucose-stimulated insulin secretion (GSIS) in a dose-dependent manner through *in-vitro* tests with INS-1E cells and primary islets by efficiently inhibiting Drak2 activity. In addition, they proved that luteolin protected β cells in part because it inhibited Drak2, reduced oxidative stress, and increased autophagy flux. Lutein treatment enhanced islet function, metabolic parameters, and glucose clearance in a mouse model of TIIDM, according to *in-vivo* investigations [75].

Luteolin and luteolin-7-O-glucoside were administered in KK-Ay mice to evaluate the effect against TIIDM. The data showed it upregulates glucose metabolism and downregulates insulin resistance, oxidative stress, and inflammation. It also reduces the liver enzymes that participate in fatty acid synthesis and gene expression, producing fat cells and inflammation [76].

The protective effect of luteolin against kidney damage caused by diabetes mellitus by its antioxidant effect was estimated in the STZ-induced Sprague-Dawley rats' diabetic nephropathy model. Different parameters were examined, such as blood glucose levels, renal function, lipid profile, antioxidant activity, and histopathology, and resulted in luteolin administration; there was substantial depilation in blood glucose levels, improvement in renal function, normalized lipid profile, enhancement of antioxidant activity, and alleviation of histopathological abnormalities related to diabetic nephropathy [77]. A similar strategy was plotted in STZ-induced diabetic nephropathy in rats conducted by Daude and team, who claimed that luteolin emerged as a potent guardian, limiting the expression of metalloenzymes such as matrix metalloproteinase-2, matrix metalloproteinase-9 and histone deacetylase-2 [78]. Furthermore, the therapeutic potential of luteolin was explored against the C57BL/6 J db/db and C57BL/6 J db/m mice model. Treatment with luteolin effectively slowed down kidney damage, decreased oxidative stress and the inflammatory response, and arrested the formation of renal fibrosis in diabetic mice. Mechanistically, luteolin exerted its beneficial effects by suppressing the activation of the STAT3 pathway and downstream inflammatory and fibrotic markers. These findings suggested that luteolin is a promising agent for ameliorating diabetic nephropathy [81].

Zn-loaded luteolin nanoparticles showed promising effects against non-alcoholic fatty liver disease induced by high-fat diet STZ in the Wistar rat model. This Zn load nano-particle acts on PI3K, AKT, and Fox01 signaling pathways, significantly improving insulin sensitivity and glucose tolerance. This drug-loaded nanoparticle has also decreased antioxidant levels and lipid peroxidation, *i.e.*, a decrease in oxidative stress. Furthermore, they ameliorate the course of non-alcoholic fatty liver disease by limiting the mechanisms involved in lipogenesis and gluconeogenesis, reducing hepatic lipids accumulation [79]. The protective effect of luteolin and their derivatives (6,8-guanidyl luteolinquinone and 6,8-guanidyl luteolinquinone chromium) was examined in high- fat diet STZ-induced rat model, and the result demonstrated that the 6,8-guanidyl luteolinquinone-chromium administration improved the liver injury and tissue inflammation involved in diabetes disease. Furthermore, RNA-sequencing analysis revealed that the compound acts *via* PPAR signaling pathways. The anti-hyperglycaemic benefits of luteolin derivatives are enhanced by their ability to control gut microbiota composition [80].

Furthermore, a multifactorial approach was conducted to explore the potential therapeutic effect of luteolin. This study revealed that luteolin treatment effectively reduced blood glucose levels, restored body weight, improved lipid profiles, modulated liver and kidney function, improved antioxidant enzymes, and diminished inflammation in animal models. The *in-silico* studies have revealed that luteolin interacts with α-glucosidase receptors and possesses anti-diabetic properties [82].

### 3.3 Naringenin

The beneficial effect of naringenin was observed in GD when it was administered in the genetic GD mice model. The results show that glucose uptake increased, improved insulin tolerance, and reduced inflammatory markers [83]. Similarly, another study was explored through *in-vitro* human skeletal, placenta, and adipose tissue and murine model *in-vivo*. *In-vitro* human tissue samples from elective cesarean sections were stimulated with TNF to mimic GD conditions. The underlying mechanism suggested that naringenin suppressed Nf κB activation, significantly improved glucose uptake, reduced inflammatory cytokines in the placenta, and elevated the expression of antioxidant mRNA [85].

In another study, the effect of naringenin found to be mitigate diabetic neuropathic pain. The author explored the study using the STZ-induced diabetic neuropathic pain in the Wister rat model. This study examined various parameters, *i.e.*, ROS matrix metalloproteinases and cytokines, which are mainly responsible for developing disease conditions, were examined. Different naringenin was administered to mice for four weeks, and the result showed that it significantly improved the behavioral changes and oxidative stress and mitigated the TNF- α, TGF-B1, and metalloproteinase-9 levels. It also suggested that combining naringenin with insulin produces a more pronounced effect than treatment alone [84].

Another study of glucose uptake and insulin stimulation potential of naringenin was examined in high glucose-treated HepG2 cells. The study assessed cell viability, glucose uptake, and the phosphorylation of AMPK and GSK3β. Results showed that naringenin enhanced glucose uptake, AMPK, and GSK3β phosphorylation, suggesting improved insulin sensitivity [86]. The promising effect of naringenin as an antioxidant and lipid peroxidation activity was explored in the fructose-STZ diabetic Sprague-Dawley rat model. Serum lipid profile, malondialdehyde, nitric oxide levels, and histological parameters were assessed. The results showed that naringenin administration significantly reduces oxidative stress and improves aortic cell histomorphology [87].

The pancreatic β-Cells protection of naringenin was investigated *in-vivo* and *in-vitro* models. MIN6 pancreatic β- cells were used *in-vitro* experiments to demonstrate that naringenin increased the expression of Nrf2 and its target genes. This resulted in protection against oxidative stress and apoptosis induced by STZ. The efficacy of this protective effect was validated *in vivo* using mouse models with diabetes and showed a significant decrease in glucose levels. Moreover, it enhances the pancreatic β-Cells activity [88]. The capacity of α glucosidase activity was explored enzymatic in both Assy and HFD-STZ-induced male albino Wistar rat models. The finding demonstrates that naringenin competitively inhibits α glucosidase, thereby delaying the breakdown of dietary carbohydrates into glucose. The in-vivo study supports the enzymatic assay; on the administration of naringenin, the test group significantly suppresses hyperglycemia [89]. Furthermore, naringenin repressed the TNF-α expression and enhanced the GLUT-4 activity in a study of HFD-STZ induced diabetic rat model to restore glucose homeostasis. This study also suggested that naringenin reduced hyperinsulinemia, improved lipid profiles, decreased membrane lipid peroxidation, enhanced antioxidant activities, and improved hepatic function markers supporting managing diabetes [90].

Antioxidant evaluation in hyperglycemia was investigated in an induced diabetic rat model. naringenin showed reduced levels of nitric oxide and increased superoxide dismutase (SOD) activity. Naringenin also exhibited anti-inflammatory effects by decreasing levels of pro-inflammatory cytokines IL-6 and TNF-α expression and ameliorating pancreatic β-cell damage, thus significantly important in treating hyperglycemia conditions [91]. Naringenin nanoparticles were prepared and used to investigate the anti-diabetic effect *in-vivo* models. The finding demonstrated that the sodium alginate-coated chitosan enhanced the naringenin efficacy and significantly lowered blood glucose levels in STZ-induced diabetic rats [95].

### 3.4 Hesperidin

The anti-diabetic activity of hesperidin has been summarized in Table **1**. The antihyperglycemic and antihyperlipidemic potential of hesperidin was examined in an STZ-induced male albino Wistar rat model. It protects against the oxidative stress induced by STZ on the pancreas. Furthermore, hesperidin improves insulin secretion, restoring the liver's glycogen levels and carbohydrate metabolic enzyme activities, increasing the activity of antioxidant enzymes, lowering dyslipidemia and markers of liver and kidney damage, and improving histological changes in the pancreas, liver, and kidney [93].

In a study of the HFD-induced obsess mice model. They treated various doses of glucosyl hesperidin and examined multiple parameters, including body weight, insulin resistance, glucose tolerance, and macrophage infiltration into adipose tissue. The results demonstrated that continuous administration of glucosyl hesperidin improved insulin resistance, glucose intolerance, hyperglycemia induced by a high- fat diet, and reduced macrophage infiltration. In contrast, the administration of glucosyl hesperidin in the short term significantly reduced the infiltration of macrophages but did not impact insulin resistance. Furthermore, in both *in-vivo* and *in-vitro* conditions, the administration of glucosyl hesperidin resulted in a reduction in the production of Monocyte Chemoattractant Protein-1 (MCP-1) [94].

Hesperidin is evaluated against inflammatory markers in the THP1 cell line. The data suggest that hesperidin significantly reduced proinflammatory cytokines such as TNF-α and IL-6, reducing the activation of NFκB and regulating TLR-mediated signaling. Additionally, hesperidin increases the SIRT3 and SIRT6 proteins known to possess anti-inflammatory characteristics [95].

### 3.5 Kaempferol

The anti-diabetic activity of kaempferol is described in Table **1**. The two main mechanisms of kaempferol against diabetes include glucose uptake and oxidative stress reduction. In a study, kaempferol investigated human skeletal muscle and obsess mice. The *in-vitro* study of skeletal muscles showed that kaempferol significantly increased glucose uptake in these cells, particularly in the presence of lipotoxic conditions induced by palmitate. The study also demonstrated that this effect is mediated by activating AMPK and PI3K/Akt signaling pathways. Additionally, *in-vivo* experiments with obese mice showed that oral administration of kaempferol improved glucose tolerance and increased the phosphorylation of AMPK and Akt in muscle tissues [96].

Moreover, kaempferol GLUT4 translocation and glucose uptake are through the JAK2-dependent pathway. This study highlighted the anti-diabetic effect of kaempferol in *in-vitro* L6 myotubes cell lines. It promotes the translocation of the glucose transporter GLUT4 to the cell membrane, facilitating glucose entry into the cells. The activation of AMPK and JAK/STAT signaling pathways mediates this process. Furthermore, kaempferol was shown to increase the phosphorylation of essential proteins involved in these pathways, specifically AMPK and JAK2, but not PI3K or its downstream targets Akt and aPKC. The findings suggested kaempferol could help maintain glucose homeostasis and potentially prevent hyperglycemia [97]. Similarly, another study was performed on L6 cell lines; in sodium palmitate-treated L6 myotubes, kaempferol significantly increases GLUT4 translocation to the plasma membrane, improving glucose metabolism. Molecular docking studies confirmed stable interactions between kaempferol and the SIRT1 protein, highlighting the role of the kaempferol moiety in hydrogen bonding [99].

In a study on protecting pancreatic-β cells cell survival, kaempferol was effective. In that study, 1NS-1E cell lines used, Kaempferol improves the cell viability, maintains the production of ATP, prevents programmed cell death, and hinders the activity of caspase-3 in β-cells and islets when exposed to elevated glucose levels. It dramatically enhances cell cAMP production and the expression of anti-apoptotic proteins Akt and Bcl-2. The study demonstrates that the protective effects of kaempferol rely on the PI3K/Akt and cAMP/PKA pathways. In addition, kaempferol enhances glucose-stimulated insulin secretion (GSIS) and insulin synthesis in β-cells and human islets when exposed to high glucose levels [98]. Furthermore, a study conducted on male C57BL/6 mice was used to investigate the effects of kaempferol on hyperglycemia induced by STZ. Kaempferol ameliorates high blood sugar levels influenced by STZ diabetic mice by reducing gluconeogenesis and enhancing glucose metabolism in the liver and skeleton [100].

### 3.6 Quercetin

The anti-diabetic potential of quercetin is summarized in Table **1**. Quercetin is isolated from the *Toona sinensis* plant to find its efficacy in diabetic rats. Alloxan and a high-fat diet are used to induce diabetes in rats, and various experiments have been conducted to evaluate their efficacy. The results show that quercetin extract reduces blood glucose and insulin levels and improves lipid and glucose metabolism. It also downregulates oxidative stress, liver enzymes, and necrosis [108].

Scientists used a diabetic model of a rat's skeletal muscle cell line. Quercetin is given to cells to treat diabetic conditions, and it is observed that it improves glucose absorption by simulating AMPK and p38MAPK pathways and upregulating the expression of GLUT 4, which improves glucose uptake [101]. When quercetin is administered in the STZ-induced Wistar Albino rat model, it decreases HbA1c, blood glucose levels, and hepatic glycogen, increasing plasma hemoglobin and antioxidant enzymes in the pancreas. Thus, it is concluded that quercetin shows an anti-hyperglycaemic effect by improving glucose metabolism and insulin secretion [102]. Researchers explored the correlation between diabetes and cancer by analyzing the impact of quercetin and glucose on the expression of mTOR and Nrf2 in HepG2 cells. Increased blood glucose increased the expression of mTOR and Nrf2, suggesting that oxidative stress plays a role in cancer progression. Dietary flavonoid quercetin reduces the expression of these molecules, thus showing an antioxidant effect and modifying the cancer-related pathways in individuals with diabetes [103].

Different activities of quercetin were investigated in C57BL/KsJ-db/db mice. The data revealed that it decreases lipide, plasma glucose, insulin resistance, and oxidative stress [104]. In TIDM, the effect of quercetin was evaluated against oxidative stress and cell apoptosis in the seminal vesicles. The quercetin alters the expression of Nrf2, T-AOC, and MDA levels, indicating antioxidant activity. Additionally, it increased the Bcl-2/Bax ratio and decreased caspase3 expression, thus showing an anti-apoptotic effect [105]. Per various studies, Quercetin was concluded to play a protective role against oxidative stress and free radical toxicity in TIIDM. In a survey of alloxan-induced Swiss albino mice, quercetin significantly decreased fasting blood glucose levels. It improved glucose metabolism by increasing the activity of hexokinase and reducing the activities of FBPase and G6Pase. Additionally, it enhanced the activities of antioxidant enzymes such as SOD, CAT, and GST. Moreover, quercetin enhanced the expression of GLUT-4 in adipocytes and skeletal muscle and restored DNA damage [106].

The anti-diabetic activity of quercetin in upregulated glucose metabolism *via* AMPK pathways was evaluated in mice (high-fat diet-fed). Quercetin stimulated the phosphorylation of AMPK in skeletal muscle, adipose tissue, and liver, resulting in enhanced translocation of GLUT4 in skeletal muscle and inhibiting adipocyte differentiation. In addition, they augmented ACC phosphorylation and elevated the expression of UCP2, CPT1, UCP1, PGC-1α, and PRDM16, thereby stimulating the conversion of white adipose tissue into brown adipose tissue. Quercetin and its glycosides reduced the expression of FAS and SREBP1 while increasing the expression of CPT1 and PPAR-α in the liver. This resulted in the modulation of lipid metabolism [107]. The effectiveness of quercetin in lowering fasting blood glucose and postprandial glucose levels was explored by Ji-Hye and their team in the STZ-induced C57BL/KsJ-db/db mice model. Quercetin did not significantly impact plasma insulin levels, but it significantly reduced maltase activity in the proximal jejunoileum. They found that it plays a role in inhibiting α-glucosidase activity [109]. The molecular mechanism and anti-hyperglycaemic activity of quercetin with GLP-1 analogs liraglutide in a high-fat/STZ-induced Albino rat model was investigated. The metabolic parameters, pancreatic tissue, oxidative stress markers, inflammation, and apoptosis were observed. Combining both quercetin and liraglutide synergically improves insulin resistance, protects B cells, and reduces oxidative stress and apoptosis [110].

Another study described the therapeutic benefits of quercetin in modulating the expression of SIRT1, which is involved in gluconeogenesis, inflammation, and TIDM. The details indicated the upregulated expression and deacetylase activity of SIRT1, which in turn activated the Akt signaling pathway, leading to enhanced phosphorylation of downstream effectors like GSK-3β and GCK and reduced levels of SREBP-1c [111]. A similar strategy was plotted to illustrate the AMPK/SIRT1/NF-κB signaling pathway in diabetic atherosclerosis rats, and on the administration of quercetin repressed the inflammatory markers NF-κB, IL-1β, oxidative stress (malondialdehyde) increased antioxidant enzyme activities (GPX, SOD, CAT) and anti-inflammatory IL-10 levels. These positive effects were linked to higher SIRT1 protein levels. They were reversed by the AMPK inhibitor, highlighting the role of the AMPK/SIRT1/NF-κB pathway in quercetin’s protective effects against diabetic atherosclerosis [112]. The super efficacy of quercetin and resveratrol was investigated in STZ-induced Sprague-Dawley rats. It resulted in a significant hypoglycaemic impact, improved glucose tolerance, and restored insulin, C-peptide, and hematological levels, highlighting its potential as a therapeutic agent for diabetes management. The histopathology confirmed the protective effect against the pancreatic tissue [113].

### 3.7 Myricetin

Table **1** summarizes the anti-diabetic potential of myricetin. The insulinotropic effect of myricetin was explored in isolated islets from ZDF rats and male Wister rats. Myricetin activates the GLUT-2 gene in INS-1 cells, increasing insulin secretion in pancreatic islets. On long-term treatment, myricetin was shown to decrease the HbA1c levels and improve the blood glucose level [114]. In another study, Myricetin restored abnormalities in the intestinal flora of mice that TIIDM triggered and was explored in a male SPF C57BL/6J mice model. Parameters such as Superoxide Dismutase, blood lipid content, and intestinal microbiota were investigated, and myricetin administration positively influenced the gut microbiota and SOD levels. This supports the myricetin compound for improving glucose metabolism and enhancing gut health in diabetic conditions [115].

Furthermore, myricetin has been shown to protect pancreatic β-cells from apoptosis by diminishing ER stress through CDK-5 and enhancing glucose-stimulated insulin secretion. This study was assessed using the INS-1 and rat islets model. The data revealed that myricetin enhances cell viability and reduces apoptosis in INS-1 cells and isolated rat islets exposed to HG conditions. It also repressed the mitochondrial dysfunction and β-cell apoptosis and interacted with CDK-5 in the docking study [116].

The anti-diabetic activity of myricetin *via* immunomodulation was explored in RAW 267.4 and Male C57BL/6J mice. The *in-vitro* assay suggested that myricetin reduced the apoptosis and ROS levels and restored the pro-inflammatory cytokines. Furthermore, the *in-vivo* study revealed that myricetin ameliorated the immunosuppressive effects by increasing the number of CD8^+^ T cells and M1 macrophages and enhancing IL-2 and IFN-γ cytokine levels [117]. The hyperglycemic activity of myricetin extract from *Syzygium malaccense* was explored against obesity-induced hyperglycemia in C57BL/6J mice. The details indicated that it enhanced antioxidant capacity and reduced oxidative stress biomarkers in kidney and liver tissues while improving glucose tolerance and serum biochemical markers, such as total cholesterol and triglycerides. The histopathological examination of the kidneys and liver showed less lipid buildup. These results point to myricetin's possible use as a medication to treat metabolic diseases and diabetes caused by obesity [119].

The anti-diabetic activity of myricetin was reported in 2015 by Kang and his team [118]. The STZ-induced diabetic mellitus Male Sprague-Dawley rats model suggested that myricetin significantly reduced serum glucose levels, glycosylated hemoglobin, and α-glucosidase inhibitors. Moreover, myricetin was reported to overexpress the β-endorphin and adropin secretion in the hyperglycaemic rat. This progression was explored in three cell lines, such as PC-12, H9c2, and HepG2 cell line medium, and for an *in-vivo* study in STZ-induced male Sprague-Dawley rats. Acute myricetin treatment enhances GLP-1 receptor expression, elevates cellular calcium, and raises cAMP levels, increasing BER and adropin secretion from the adrenal gland. Chronic myricetin administration further boosts adropin levels, even in the absence of the adrenal gland, suggesting direct effects on hepatocytes [120]. Anti-DPP4 inhibitor and GLP-1 agonist activity of myricetin was studied in HFD-STZ induced male Wistar rat model. The GLP-1 agonist leads to enhanced insulin secretion in diabetic rat models. Myricetin also boosts antioxidant enzyme activities, mitigates inflammation by downregulating NLRP3 inflammasome, PYCARD, and caspase-1, and lowers circulating IL-1β levels [121].

### 3.8 Taxifolin

The anti-diabetic potential of taxifolin has been summarized in Table **1**. In a study by Gurumayum and team, they explored the anti-diabetic properties of *Osbeckia nepalensis* Hook. The extract demonstrated the ability to enhance glucose absorption in CC1 hepatocyte cell culture studies and modulate the hepatic gluconeogenic expression such as AMPK, G6Pase, PGC1α, FOXO1, HNF4 α, and PEPCK without causing any harmful effects. Through additional separation, it was determined that the n-butanol fraction exhibited the highest activity level. These experiments conducted on STZ-induced diabetic rats showed that BuSFr1 effectively reduced blood glucose levels, enhanced insulin sensitivity, and mitigated liver damage [123]. Furthermore, the authors conducted a study to examine the impact of Taxifolin succinate on diabetic rats. The researchers developed diabetes in Wistar rats by administering STZ, measuring body weight and food consumption, fasting blood glucose levels, performing OGTT, and evaluating the antioxidant effect. The result suggests that taxifolin administration increases blood glucose levels and OGTT outcomes and protects pancreatic and liver tissues [124].

Scientists carried out *in-vivo* and *in-vitro* studies of taxifolin against diabetes. The L6 myotubes and KK-Ay/Ta mice are used for *in-vitro* and *in-vivo* models. In the *in-vitro* model, taxifolin stimulated glucose uptake in muscle cells by activating the PI3K/Akt and AMPK pathways. This resulted in increased translocation of the glucose transporter GLUT4 to the plasma membrane. Administration of taxifolin in mice with TIIDM demonstrated that it reduces blood glucose levels, uric acid levels, and insulin resistance. Thus, taxifolin is beneficial in TIIDM conditions in rats and can be used further [125].

Moreover, the anti-hyperglycemia activity of taxifolin was evaluated in molecular dynamics simulations and an alloxan-induced diabetic rat model. *In-silico*, taxifolin was found to have more affinity than acarbose towards α-amylase. The data shows that it improved levels of enzyme α-amylase and lipase and had a beneficial effect on postprandial blood glucose levels and lipid levels in a dose-dependent manner. Taxifolin is more advantageous in improving liver function and antioxidant levels than acarbose. The results indicate that taxifolin has inhibitory activity against α-amylase, which helps manage diabetes [126].

In a study, taxifolin reduces oxidative stress and inflammation induced by alloxan in a diabetic retinopathy rat model. Results showed that taxifolin significantly reduced malondialdehyde, IL-1β, and TNF-α levels while increasing total GSH levels in the treated rats compared to the diabetic controls. As a result, it is indicated that taxifolin lowers inflammation and improves antioxidant defense [122].

## 4 FLAVONOIDS IN DIABETES TREATMENT: BENCH TO BEDSIDE-CLINICAL INSIGHTS

Many flavonoids that showed effectiveness in animal research for diabetic treatment have now been tested in people to see if they are safe and effective. The use of flavonoids in human study has been the subject of 18 separate clinical investigations. Nevertheless, 5 distinct flavonoids used in clinical treatment for patients are documented in this research, each searched separately. The summary of flavonoids used in patients is given in Table **2**.

## 5 REVOLUTIONIZING DIABETES TREATMENT: NOVEL DRUG DELIVERY SYSTEM LEADING THE WAY

### 5.1 Microparticulate and Diabetes

Microparticulate is a type of particulate drug delivery system where the drug is encapsulated with particles with a spherical shape and a diameter ranging from 10 to 1000 micrometers (μm) size [127, 128]. The microparticulate system is engineered to target specific sites with higher concentrations and accept a variety of routes. It protects the drug from degradation, such as enhancing insulin stability and keeping it unchanged in the gastrointestinal tract. The microparticulate systems can deliver the drug over an extended period. It minimizes the dose frequency due to a larger payload than a nanoparticulate system. It also overcomes the dumping dose [129]. Microparticulate systems facilitate the simultaneous delivery of various medications or therapeutic agents, enabling combination therapy in managing diabetes. Microparticulate systems can deliver a wide range of drugs, including those that are soluble or insoluble in water, as well as proteins, peptides, and nucleic acids. This makes them a versatile platform for managing diabetes [130, 131].

### 5.2 Nanotechnology and Diabetes

Nanotechnology involves using nanoparticles and other nanoscale materials to improve the delivery of therapeutic levels of agents, such as drugs and other molecules, to the target sites within the body. The term refers to the precise modification of atom, molecule, and supermolecule scales using different physical and chemical properties methodologies. The nanoparticle size ranges from 1 to 100 nanometers (nm) [132]. In nanotechnology, the drug is uniformly dispersed in the matrix system, or the drug molecule is incorporated with a unique polymeric substance. These nanospheres/nanoparticles have a vast surface area and quickly reach the target sites, activating and improving the drug's bioavailability, safety, and efficacy [133]. Literature suggests this technology can improve absorbance 15 to 250 times more than macroparticles [134]. This nanotechnology also facilitated the closed-loop insulin delivery strategy (self-regulated insulin delivery system), where the nanoscale carbon senses the blood glucose level and releases the accurate insulin dosing in the body [135].

### 5.3 Transdermal Delivery System

The skin, the largest organ in the body and gets around thirty percent of the total blood supply, was recognized as a pathway for delivering systemic medications in the late 20^th^ century [136]. The non-invasive transdermal administration technique has gained popularity among patients because of its higher compliance than conventional oral and parenteral delivery methods. A transdermal delivery system involves various drug delivery techniques across the skin. An iontophoretic technique is applicable for providing the charge or ion drug, here low electrical current to the skin, which drives the charged drug molecule [137, 138]. The microneedle array technique allows the medicine to permeate the skin utilizing tiny needles that create micropores in the stratum corneum. The Sonophoresis technique uses low-frequency ultrasound to break up the stratum corneum, which makes it easier for the drug to get into the skin. The nanoparticle and liposome carrier-based nanofibers for regulated drug delivery increased bioavailability and therapeutic effectiveness. Transdermal patches bypass first-pass metabolism and concentration-related side effects, delivering medication straight to the systemic circulation over a controlled period by a simple delivery method [139].

### 5.4 Gene Therapy

Precision medicine is a critical aspect of treating diabetes diseases based on the individual genes, environment, and lifestyle [140]. As per the European Medicines Agency (EMA), gene-based therapy is a technique that inserts recombinant genetic material into humans to regulate gene defects and therapeutic effects and exaggerate therapeutically beneficial proteins [141, 142]. Gene-based therapy is divided into two categories: germ-line gene therapy, in which the recombinant nucleic acid transfers into the next generation, and somatic gene therapy, in which the modified gene or transcription factor will not transfer into the next generation. Multiple genes have been implicated in the pathogenesis of diabetes mellitus. Gene-based therapy involves meticulously identifying the pivotal gene or genes underlying the disease. Subsequently, the synthesis of a healthy or therapeutic gene ensues. Following synthesis, meticulous vector selection precedes vector transfer into the targeted tissue. Finally, the integrated targeted gene assumes its function and is expressed therein [143].

### 5.5 Stem Cell Therapy

Stem cell therapy is a promising and rapidly evolving field with potential applications in various medical conditions. Stem cells are precursor cells found in different tissues that can differentiate into multiple cell types, making them valuable for therapeutic purposes. Stem cell therapy holds great potential for the treatment of diabetes mellitus, particularly TIDM, which is characterized by the loss of pancreatic β cells, leading to insulin deficiency and hyperglycemia. The shortage of pancreas and islets from human organ donors, complications associated with transplantations, high cost, and limited procedural availability have led to the exploration of stem cell therapy as a promising alternative [144, 145]. The primary objective of stem cell therapy for diabetes is to derive insulin-producing cells (IPCs) from various precursor cells and utilize diverse strategies to optimize the therapeutic advantages of stem cells. There are many IPCs generating cells are available and used clinically, such as mesenchymal stem cells (MSCs), human pluripotent stem cells (hPSCs), human embryonic stem cells (hESCs), and human induced pluripotent stem cells (hiPSCs) [146]. MSCs are a potential cell therapy because of some properties such as easy isolation, multi-differentiation potential, and immunomodulatory effect. However, hPSCs offer an unlimited source of pancreatic cells that can secrete insulin in response to high blood glucose levels. As per the literature, BM-HSCs are an essential resource for stem cell transplantation in TIDM. In addition, MSC therapy and bone marrow mononuclear cell (BM-MNC) therapy are good sources for stem cell transplantation for TIDM [147].

## 6 DISCOVERING FLAVONOIDS: PHARMACOLOGICAL INSIGHTS, CONSTRAINTS, AND FUTURE DIRECTIONS

Diabetes is a prevalent and challenging health condition, with a projected increase in global prevalence. Due to the increasing burden, diabetes management is now of concern. Several modern medicines available in the market for managing diabetes are effective. Still, medicines often have a lot of possible side effects that can be tough for patients to deal with thiazolidinediones associated with anemia, edema, myocardial injury, and weight gain [148]. DPP4 inhibitors may cause upper respiratory tract infections and nasal pharyngitis [149]. Other drugs, such as SGLT 2 inhibitors, may cause renal failure, genitalia infection, and breast and bladder cancer [138, 139] and α-glucosidase inhibitors may cause gastrointestinal issues like diarrhea and stomachache [150, 151]. To address the growing diabetes epidemic, reduce side effects, improve patient outcomes, and provide effective treatment options, several modern novel treatments are being explored.

Diabetes is a multifactorial disease, with multifaceted mechanisms of action required for the treatment of diabetes and complications rather than focusing solely on blood sugar control. Effective and safer medications can be derived from various natural sources, including plants, animals, microbes, and marine life. The traditional management of diabetes uses medicinal plants, with a focus on addressing not only diabetes but also its sequelae and fewer side effects. They are practical, less expensive, and readily available compared to conventional medications.

Plant-derived flavonoids have a variety of pharmacological properties that make them helpful in treating and managing diabetes and other diseases [152, 153]. Flavonoids have been reported to inhibit activating kinases and carbohydrate-metabolizing enzymes, thereby influencing all metabolic pathways of proteins, carbohydrates, and lipids. They can also intervene in the insulin-signaling pathway, oxidative stress, and modulate inflammatory responses. Many studies have been plotted to prove flavonoids have potent anti-diabetic activity. The primary mechanism includes i) Glucose uptake enhancement and insulin sensitivity, ii) β- cell protection and insulin secretion, iii) Oxidative stress and inflammatory modulation, iv) Glucose metabolism and postprandial hyperglycemia, and v) Gut microbiota modulation.

Kaempferol, quercetin, and taxifolin have demonstrated the ability to improve glucose uptake by activating essential signaling pathways, including AMPK, PI3K/Akt, and p38 MAPK. Kaempferol enhances glucose uptake in muscle tissues during lipotoxic conditions by stimulating the AMPK and PI3K/Akt signaling pathways. Quercetin boosts glucose absorption and increases the expression of GLUT4 in myotubes. This is achieved through the activation of AMPK and p38 MAPK pathways. Taxifolin also enhances the translocation of GLUT4 through the PI3K/Akt and AMPK pathways, indicating a coordinated effect in improving insulin sensitivity and maintaining glucose homeostasis.

Flavonoids protect pancreatic-β cells, such as kaempferol, myricetin, apigenin, and naringenin. Apigenin ameliorates ER-stressed-induced apoptosis by inhibiting TXNIP expression, whereas naringenin protects the β-cells from apoptosis induced by oxidative stress. The synergistic effect can significantly improve β-cell function and insulin secretion, where kaempferol Improved the survival cell count by activating PI3k/AKT and Camp/Ph pathway and increased insulin production. Myricetin upregulates the gene expression, *i.e.*, The glut-2 gene promotes insulin release and downregulates β-cell death.

Oxidative stress and inflammation are very prone to diabetes. Flavonoids such as quercetin, myricetin, taxifolin, luteolin, naringenin, and hesperidin have exact protective effects. Myricetin decreases ROS expression and inflamma- tory cytokines, thereby restoring immune homeostasis. Quercetin activates the antioxidant enzymes that ameliorate the glycosylated hemoglobin. The results are additionally confirmed by Hesperidin's function in diminishing inflammation *via* TLR-mediated pathways and augmenting the expression of SIRT3 and SIRT6.

Flavonoids affect the carbohydrate metabolism. The finding shows that apigenin acts as an inhibitor of α-glucosidase and taxifolin augmenting α-amylase, resulting in countering postprandial hyperglycemia. Microbiota, essential for the regulation of metabolism, lutein and myricetin also benefit gut microbiota and upregulate the antioxidant expression.

Preclinical studies consistently demonstrate that flavonoids possess significant potential as potent anti-diabetic agents. Nevertheless, the advancement of implementing these encouraging outcomes into clinical trials has been limited, with only 5 trials with hesperetin, kaempferol, and quercetin carried out thus far. There are several limits, even though numerous pathways have been discovered to explain how flavonoids alleviate diabetes. The primary challenges impeding the clinical application include:

- Poor stabilityL: flavonoids are highly susceptible to oxidation, heat, and light primarily due to the presence of the unsaturated double bonds and the presence of the hydroxyl (-OH) group in their structure [154]. Quercetin and myricetin are particularly prone to oxidative degradation, leading to toxic byproducts or less active [155, 156]. Similarly, apigenin and luteolin are degrading rapidly in alkaline conditions [157, 158]. Heat sensitivity also contributes to instability, as demonstrated by the reduced efficacy of naringenin at elevated temperatures [159]. The difficulty in developing and using flavonoid medicines is due to their complicated structures, many active sites, and lack of selectivity and specificity. Developing a structure-activity relationship and drug design using a fragment-based approach is crucial to improving the poor stability of flavonoids.
- Bioavailability: bioavailability is mainly hindered by low solubility, fast metabolism in the liver, and rapid elimination from the body. Most flavonoids are ingested, and in intestinal cells, the extra free hydroxyl groups of flavonoids are rapidly glucuronidated and sulfated before being effluxed *via* transporters. When the amount of hydroxyl groups rises, this process decreases gastrointestinal permeability, which results in uneven and poor oral absorption of these flavonoids [160]. Thus, to improve oral bioavailability, absorptions and permeability of flavonoids are required. In addition, the presence of hydroxyl moieties and unsaturated double bonds in flavonoids are also sensitive to body physiological conditions (*i.e.*, pH and digestive enzyme) that hamper biotransformation and damper systemic circulation. Furthermore, the presence of double bonds in the 2 and 3 positions of flavonoids prevented the solvent from entering the molecule, which led to poor water solubility [161]. Therefore, further exploration is needed to improve solubility. Nevertheless, existing formulations such as capsules, tablets, oral solutions, and granules have failed to overcome their poor solubility and inconsistent bioavailability. Therefore, modern advancements like bio-enhancers, derivatization, encapsulation, and nanotechnology are required to support the clinical utilization of flavonoids.
- Inconsistent efficacy in human trials: transition from preclinical settings to clinical settings requires extensive investigation of numerous factors to ensure the safety and effectiveness of putative therapies. The therapeutic impact of flavonoids must be evaluated using plausible criteria, which must be established. So, further investigation should verify the targeted pathways and underlying processes in humans, as animal models frequently do not accurately reflect human physiology. Due to the inherent heterogeneity in human responses, validating the efficacy of preclinical investigations with thorough clinical trials are essential. Another important consideration is safety. Human outcomes may not be predicted by preclinical safety profiles. Thus, extensive toxicity and side effect evaluations in clinical trials are required. Furthermore, ascertaining the suitable therapeutic dosage is crucial as human dosages may not always correspond as doses effective in animals. These difficulties sometimes cause a delay in the translation of preclinical results into clinical practice, highlighting the necessity of thorough clinical research to guarantee the safe and effective use of flavonoids in the treatment of diabetes.
- Lack of standardization: flavonoid concentrations vary greatly among plant species and extraction techniques, and this variability in flavonoid supplement standards makes it difficult to achieve reliable therapeutic effects. Because of the varying quantities of active chemicals caused by this diversity in quality and purity, it is challenging to guarantee consistent impact and safety. Lack of purification techniques might add contaminants, worsening effectiveness even further. The absence of standardization also adds complexity to the clinical trial findings. It bars the development of defined dosage recommendations, therefore questioning challenges for healthcare practitioners in confidently dispensing flavonoid supplements. To overcome these barriers, it is crucial to establish standardized extraction and formulation techniques and rigorous quality inspection and regulatory frameworks to guarantee the uniformity and reliability of flavonoid supplements in therapeutic applications.

Recent innovative technologies are being developed to mitigate the rise of this problem. Microparticulate systems and nanotechnologies protect flavonoids from degradation, improve delayed absorption, enhance bioavailability, and improve stability. Likewise, Transdermal patches bypass the flavonoids from the digestive system and strengthen bioavailability and controlled release to optimize the therapeutic effectiveness. Furthermore, the combination of stem cell therapy with flavonoids improves the treatment of diabetes by enhancing stem cell viability, cell proliferation, and function [162]. This synergistic approach offers a means to improve glycaemic control and tissue repair. These novel methods are essential for overcoming the pharmacokinetic constraints of flavonoids, enabling the exploitation of their complete therapeutic potential (Fig. **4**).

## CONCLUSION

Emerging data has indicated that flavonoids exhibit considerable potential as anti-diabetic agents. Hence, further meticulous preclinical research is necessary to determine the safety and effectiveness of flavonoids to support the requirement of appropriate clinical studies in this relation. Nevertheless, evaluating the safety profile for plant products containing flavonoids is challenging because of inadequate evaluation methodologies for isolating and characterizing these compounds despite their extensively documented use in clinical illness treatment. Therefore, it is necessary to carry out rigorous studies to determine the safety and effectiveness of flavonoids. Nevertheless, some pharmacopoeia have published comprehensive monographs on testing plant products. Additional studies should be undertaken in the way of clinical studies to validate their effectiveness and utility in human subjects.

## Figures and Tables

**Fig. (1):**
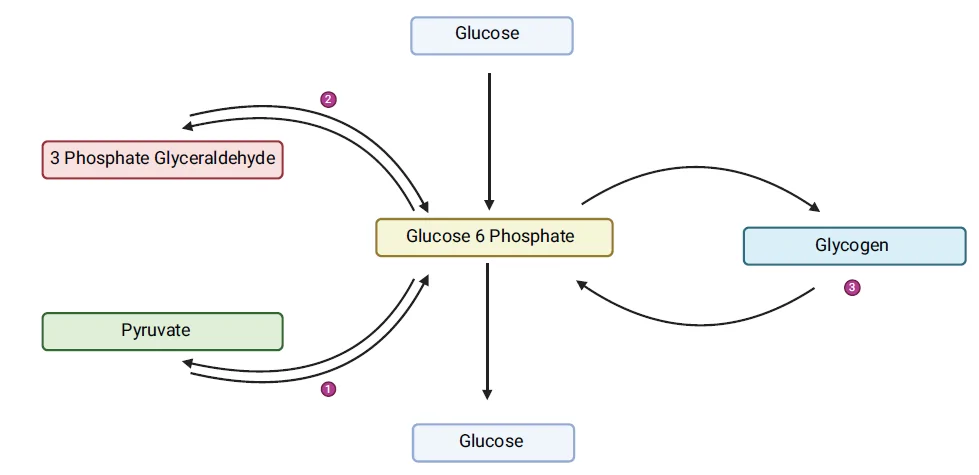
Physiology of energy metabolism. Physiology of energy metabolism. 1) Triphosphate pathway by which glucose-6 phosphate is converted to pyruvate, 2) Pentose phosphate shunt pathway by which glucose-6 phosphate is converted to 3-phosphor glyceraldehyde. 3) Glycogen is synthesized from uridine diphosphate-glucose, which is initially converted from glucose-6-phosphate.

**Fig. (2):**
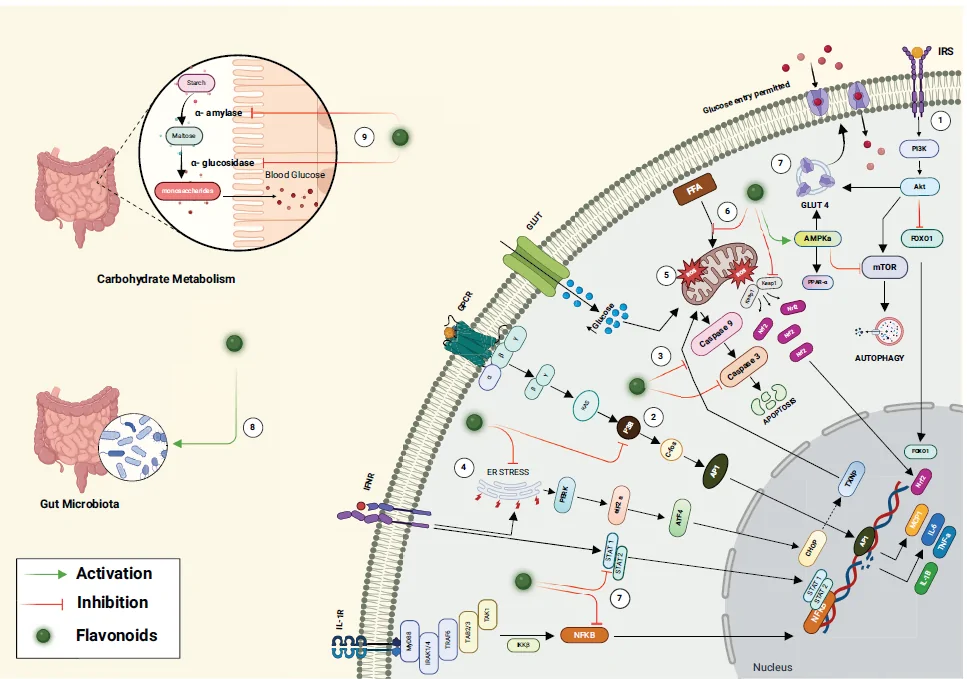
Role of flavonoids in the management of diabetes. 1. Flavonoids activate AMPK, PI3K/Akt pathway. 2. Upregulate the GLUT-4 expression. 3. Flavonoids normalized the FFA-induced oxidative stress. 4. flavonoids intervene in the keap 1 to bind Nrf2 and upregulate the antioxidant activity. 5. flavonoids suppress TXNIP and inhibit the apoptosis induced by oxidative stress. 6. Flavonoids inhibit the p38 expression. 7. downregulated the ER stress. 8. Flavonoids inhibit NF-Κb and STAT 1/STAT 2. 9. Beneficial impact on gut microbiota. 10. Flavonoids hinder α-amylase and α-glucosidase.

**Fig. (3):**
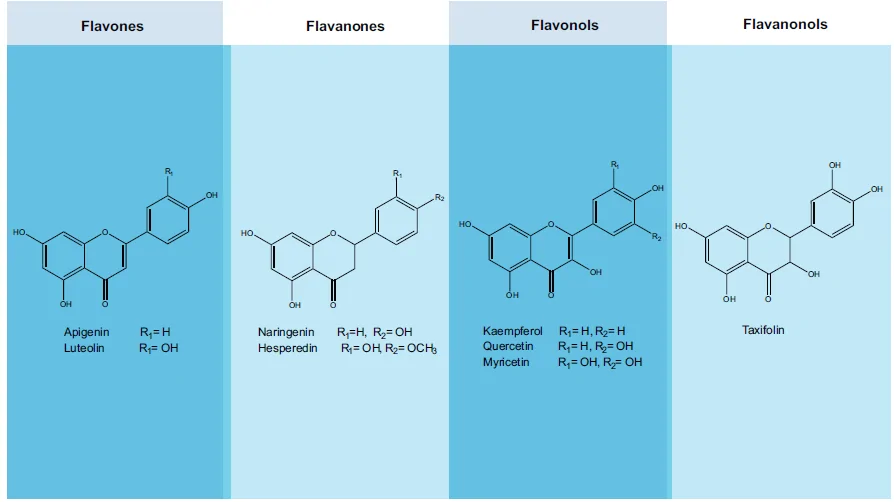
The structure of major flavonoids.

**Fig. (4):**
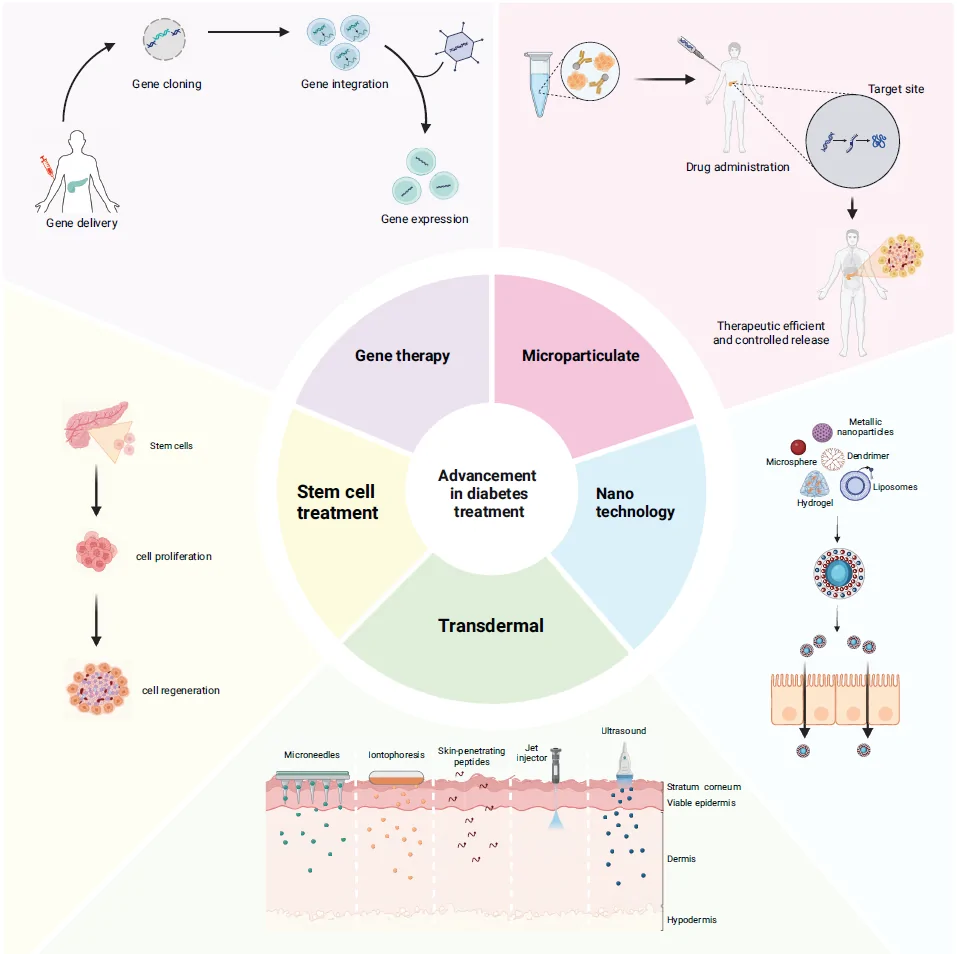
Cutting-edge strategies in diabetes treatment.

**Table 1:** Summary of the research conducted on Flavonoids.

-	**Scientific Evidence**		**Outcomes**	**References**
	***In-Vitro***	***In- Vivo***		
**Flavones**				
Apigenin	HepG2 cell line	-	Upregulates glycogen synthesis and glucose consumption by stimulating GLUT 4.	[67]
	INS-1 β-cell line	-	It shows an antidiabetic effect by releasing insulin stimulated by glucose on β-cells, reducing apoptosis.	[68]
	α-glucosidase inhibitory assays	-	Possess inhibiting α -glucosidase.	[69]
	α-glucosidase inhibitory assays	High-fat diet- STZ induce mice model	Possess α-glucosidase inhibiting properties.	[70]
	-	C57BL/6J mice	Reduce oxidative stress *via* stimulating Nrf2/HO-1 and AMPK/PI3K/Akt/eNOS pathways.	[71]
	-	Streptozotocin-induced diabetic rats	It modulates GLUT4 and CD38 to lower blood glucose level.	[72]
	-	streptozotocin-induced type 1 diabetes in albino Wister rats	It improve Drp1 expression.	[74]
Luteolin	INS-1E cell lines	-	Protect β-cells by decreasing apoptosis and oxidative stress.	[75]
	-	KK-Ay mice	Enhance the glucose metabolism and reduce insulin resistance.	[76]
	-	Streptozotocin-induced diabetic in Sprague-Dawley rats.	Increases antioxidant level	[77]
	-	streptozotocin-induced diabetic nephropathy	Inhibit metalloenzymes (HDAC-2, MMP-2, and MMP- 9) and reduce proinflammatory cytokines and oxidative stress.	[78]
	-	High fat diet-STZ-induced diabetic Wistar rats	Zinc-loaded luteolin nanoparticles improve hepatic conditions by regulating insulin resistance and oxidative stress.	[79]
	-	High-fat diet-STZ-induced diabetic rats	Improve metabolism of glucose by regulating the PPAR pathway.	[80]
	-	C57BL/6 J db/db mice	It protects against diabetic nephropathy by reducing inflammation and oxidative stress *via* the STAT3 pathway.	[81]
	α-glucosidase inhibitory assays	STZ-induced diabetic in Wister rats	Reduce oxidative stress and inflammation.	[82]
**Flavanones**				
Naringenin	-	C57BL/KsJ mice	Improve insulin sensitivity by AMPK pathway and downregulate inflammatory cytokines.	[83]
	-	STZ-induced diabetic in Wister rat models	Improve hyperglycemic condition and diabetic neuropathic pain by altering the MMP-9 pathway.	[84]
	Human placenta, visceral adipose tissue, and skeletal muscle	Murine model of pregnant heterozygous mice	Improve insulin sensitivity and reduces oxidative stress and inflammatory cytokines.	[85]
	HepG2 cell lines	-	Enhance the glucose uptake by increasing phosphorylation of the AMPK pathway.	[86]
	-	Fructose-STZ diabetic rat model	Improve hyperglycemia and oxidative stress.	[87]
	MIN6 cells	STZ induced diabetes	Protect pancreatic β-cells by activating Nrf2.	[88]
	-	High-fat diet-STZ induced in albino Wister rats	Reverse the hyperglycemia by inhibiting α-glucosidase.	[89]
	-	High-fat diet-STZ induced in albino Wister rats.	Improve blood glucose, lipid, oxidative stress, inflammation, and modulated GLUT-4	[90]
	-	Alloxan-induced diabetes in albino Wister rats	Altered the inflammation and NOS expression.	[91]
	-	STZ-induced diabetes rats	Nanoparticles of naringenin improve blood glucose levels, lipid metabolism, and oxidative stress.	[92]
Hesperidin	-	STZ-induced albino Wister rats	Improved hyperglycemic and dyslipidemia conditions.	[93]
	-	High-fat diet-induced obese mice.	Reduced the macrophage infiltration and MCP-1 expression.	[94]
	THP1 cells	-	Suppress the expression of TLR/MyD88/NF-Kb.	[95]
**Flavonols**				
Kaempferol	C2C12 cells	C57BL/6 mice	Upregulated the glucose uptake in skeletal muscle by the AMPK/Akt pathway.	[96]
	L6 myotubes	-	Suppresses hyperglycemia by altering the JAK2-dependent pathway.	[97]
	L6 myotubes	-	It protects the β-cells of the pancreas and increases insulin secretion, thus blood glucose level.	[98]
	Skeletal muscle (L6) cells	-	Improved blood glucose and insulin sensitization by activating SIRT1.	[99]
	-	STZ-induced diabetes in C57BL/6 mice	Increased glucose metabolism and decreases hepatic gluconeogenesis.	[100]
Quercetin	L6 myoblasts		Improved glucose uptake by the AMPK pathway.	[101]
	*In vitro* model	STZ-induced diabetic Wister albino rats	Reduced blood glucose and glycated hemoglobin.	[102]
	HepG2 cells	-	Reduced blood glucose by reducing oxidative stress.	[103]
	-	C57BL/KsJ-db/db mice	Improved upregulation of glucose, lipid, and oxidative stress.	[104]
	-	STZ-induced diabetes rats	Inhibited apoptosis-induced oxidative stress *via* inhibiting Nrf2.	[105]
	-	Alloxan-induced diabetes in Swiss Albino mice	Improved oxidative stress and DNA damage.	[106]
	-	High-fat diet mice	Improved hyperglycemia by activating the AMPK pathway.	[107]
	-	High-fat diet-alloxan-induced diabetes in mice	Inhibited p65/NF-κB and ERK1/2/MAPK pathway.	[108]
	-	STZ induced C57BL/KsJ-db/d mice	It maintains blood glucose levels during fasting.	[109]
	-	High-fat diet-STZ induced diabetes in Albino rats	Improved hyperglycemia insulin sensitivity and reduces the expression of oxidative stress, inflammation.	[110]
	-	STZ-induced diabetes in Sprague-Dawley rats	Upregulated the metabolism of glucose and lipids by activating SIRT1 and Akt Signaling.	[111]
	-	High-fat diet diabetic- atherosclerosis rats model	Improved atherosclerosis associated with diabetes by reducing oxidative stress and inflammation *via* AMPK/SIRT1/NF-κβ signaling pathway.	[112]
	-	STZ-induced diabetes in Sprague-Dawley rats	Improved dyslipidemia, insulin level, and serum glucose level and also improved the structure of pancreatic β-cells.	[113]
Myricetin	-	ZDF rats	It maintained the level of glucose *via* the GPCR agonist.	[114]
	-	High-fat diet-STZ induced in SPF C57BL/6J mice.	Improved blood glucose and symptoms associated with diabetes by maintaining intestinal microflora.	[115]
	INS-1 cells	-	Restricted β-cells from apoptosis induced by upregulated blood glucose by inhibiting endoplasmic stress.	[116]
	RAW 264.7 cells	High-fat diet supplement in C57BL/6J mice	Normalized blood glucose and lipid also decrease pre-diabetic condition.	[117]
	α-glucosidase inhibitory assay	STZ-induced diabetes in Sprague-Dawley rats	It prevented hyperglycemia by inhibiting α-glucosidase.	[118]
	-	High-fat diet in C57BL/6J mice	Improved hyperglycemia and oxidative stress.	[119]
	PC-12, H9c2 cells, HepG2 cells	STZ-induced diabetes in Sprague-Dawley rats	Improved blood glucose levels by activating GLP-1.	[120]
	-	High-fat diet-STZ induced diabetes in Wister rats	Improved the level of insulin by decreasing the DPP-4 inhibitor and upregulating GLP-1.	[121]
**Flavanonols**				
Taxifolin	-	Alloxan-induced diabetic retinopathy rat.	MDA, IL-1β, and TNF-α levels were significantly decreased.	[122]
	CC1 hepatocytes	STZ-induced diabetic male Wistar rats	Enhanced the uptake of glucose and downregulate the G6Pase and PEPCK.	[123]
	-	STZ-induced diabetes mellitus type 1	The fasting blood glucose level of the diabetic animals decreased, and the protective effect on the pancreas and liver.	[124]
	Cultured L6 muscle cells (myotubes)	Type 2 diabetic (T2D) model KK-Ay/Ta mice	Suppresses the hyperglycemic level.	[125]
	-	Alloxan-induced diabetic animal model	Inhibitor of α-amylase.	[126]

**Table 2:** Summary of the clinical trials conducted on flavonoids.

**Flavonoids in Diabetes ** **Treatment: Bench to Bedside- Clinical Insights**	**Trail ID**	**Title**	**Disease Condition**	**Start Date- End Date**	**Phase**	**Country**	**Type of Formulation**	**Intervention**	**Age Group**	**References**
Hesperetin	NCT05243238	Hesperidin-Diosmin Effect on Metabolic Syndrome and Diabetic Neuropathy Among Patients With Type 2 Diabetes Mellitus	Metabolic syndrome and diabetic neuropathy	March 2020 - Nov 2021	-	Egypt	-	Dietary supplement	18-70	[128]
	NCT03737422	The Effects of Hesperidin and Flaxseed on Prediabetes	Prediabetes	Jan 2018 - Dec 2018	-	Iran	Capsule	Dietary supplement	18-70	[129]
Kaempferol	NCT02760017	Bauhinia Forficata in Diabetic Patients	TIIDM	April 2016 -Sept 2017	-	Brazil	Capsule	Dietary supplement of Bauhinia forficata 200 mg of plant extract	18-75	[130]
Quercetin	NCT00065676	Investigating the Use of Quercetin on Glucose Absorption in Obesity and Obesity with Type 2 Diabetes	Obesity and diabetes	April 2010 - Aug 2021	2	United States	-	1-2 gm of quercetin given	18- 65	[131]
	NCT01839344	Effects of Quercetin on Blood Sugar and Blood Vessel Function in Type 2 Diabetes.	TIIDM	May 2013- March 2015	2	United States	Capsule	Dietary supplement quercetin 250 mg capsule, oral single dose of 2000 mg	18-75	[132]

## LIST OF ABBREVIATIONS

TIDM: Type-I Diabetes
2AG: 2-Arachidonoylglycerol
3 SOD: Superoxide Dismutase
AEA: Anandamide
CAT: Catalase Extracellular Signal-regulated Kinase
GD: Gestational Diabetes
Grb2: Growth Factor Receptor Bound
HPA: Hypothalamus Pituitary Adrenal
IKKβ: Serine Kinases IκB Kinase β
IL: Interleukin
IR: Insulin Receptor
IRS: Insulin Receptor Substrate
JNK: Jun N-terminal Kinase
MAPK: Mitogen-activated Protein Kinase
MAPK/Erk: Mitogen-activated Protein Kinase/Extracellular Signal-regulated Kinase
mTOR32: Mammalian Target of Rapamycin 32
mTORC1: mTOR Complex 1
NEFA: Non-esterified Fatty Acid
Nrf2/HO-1: Nuclear Factor Erythroid 2-related Factor 2/ Heme Oxygenase-1
PI3K/Akt: Phosphoinositide-3-Kinase-Protein Kinase
ROS: Reactive Oxygen Species
S6K: Ribosomal Protein S6 Kinase
STAT3: Signal Transducer and Activator of Transcription
TIIDM: Type-II Diabetes
TNF-α: Tumour Necrosis Factor Alpha
TXNIP: Thioredoxin-interacting Protein
